# Unique Constant Phase Element Behavior of the Electrolyte–Graphene Interface

**DOI:** 10.3390/nano9070923

**Published:** 2019-06-27

**Authors:** Jianbo Sun, Yuxin Liu

**Affiliations:** Lane Department of Computer Science and Electrical Engineering, West Virginia University, Morgantown, WV 26506, USA

**Keywords:** electrolyte–graphene interface, constant phase element, frequency response

## Abstract

We report a unique constant phase element (CPE) behavior ($\frac{1}{Z}=Q_{0}{\left(j\omega \right)}^{\alpha }$) of the electrolyte–graphene interface with both $Q_{0}$ and α showing dependence on the gate voltage. The frequency response of the electrolyte–graphene interface was studied using electrochemical impedance spectroscopy (EIS). The result suggests that (1) the electrolyte–graphene interface should be characterized as a CPE (α < 1), rather than an ideal capacitor; and (2) both $Q_{0}$ and α show ambipolar dependence on the applied voltage. We speculate that the CPE behavior of the electrolyte–graphene interface arises from the charged impurities on the substrate and the defects in the graphene lattice, which could introduce inhomogeneity of local density of states (DOS). The low density of states of graphene makes α sensitive to these local DOS near the Dirac point, and thus showing dependence on the gate voltage. Measurement of the electrolyte–graphene interface capacitance based on multi-frequency capacitance-voltage (CV) profiling was demonstrated, and the extraction of the carrier mobility was performed. The study could lead to a more accurate understanding of the capacitive behavior of the electrolyte–graphene interface, which is instructive for the design and analysis of devices involving the electrolyte–graphene interface for nanoelectronics and bioelectronics applications.

## 1. Introduction

The electrolyte–graphene interface plays a critical role in many promising applications of graphene, such as supercapacitors [1,2], biosensors [3,4], electrodes [5], etc. It is, therefore, of considerable significance to have an accurate and more in-depth understanding of its properties. Of particular interest is its capacitance, which is a critical parameter that determines a device’s performance. The electrolyte–graphene interface can be modeled as two capacitors in series connection: the capacitance of the electrical double layer (EDL) and the quantum capacitance of graphene. Theoretical calculations indicate that the quantum capacitance of graphene dominates the total interfacial capacitance for few-layer graphene at low gating potentials, which originates from the low density of states (DOS) in graphene at low energy levels [6,7]. At potentials >1 V, the total capacitance is limited by the universal capacitance of the EDL due to the dielectric saturation of water and the steric effects of the ions at the electrolyte–graphene interface [7]. Experimentally, Xia et al. [8] first measured the electrolyte–graphene interfacial capacitance and extracted the quantum capacitance of graphene; the measured interfacial capacitance was shown as a V-shape dependence on gate voltage with a non-zero minimum at the Dirac point. Several other reports have recently demonstrated similar voltage-dependence of the interfacial capacitance of graphene in various electrolytes, such as ion-gels [9] and aqueous electrolytes [10,11,12].

These studies have revealed the voltage-dependence of the electrolyte–graphene interfacial capacitance; however, the measurements in most of these studies were performed at fixed frequencies, or within limited frequency ranges, thus failing to display the complexity of the capacitive behavior of the electrolyte–graphene interface. Typically, the liquid–solid interface does not behave as an ideal capacitor but is usually considered as a constant phase element (CPE), with the impedance (Z) in the form as follows [13]:

(1) $$Z=\frac{1}{Q_{0}{\left(j\omega \right)}^{\alpha }}$$

in which $Q_{0}$ has the numerical value of the admittance at ω = 1 rad/s. The impedance of an ideal capacitor has a phase angle of −90°; the phase angle of the impedance for a CPE is −90°·α (0 < α < 1). The frequency dispersion of the measured interfacial capacitance has been observed in several previous reports [10,14,15]. In Du et al.’s study [14], two CPE were used in the electrolyte–graphene interface model, which can well fit the experimental results. In addition, the relatively high sheet resistance of graphene could also lead to the frequency dispersion of the measured interfacial capacitance due to the distribution of resistive and capacitive circuit elements along with the electrolyte–graphene interface [16].

In this work, we studied the frequency response of the electrolyte–graphene interface using electrochemical impedance spectroscopy (EIS), and revealed a unique CPE behavior of the electrolyte–graphene interface, showing both $Q_{0}$ and α are dependent on the gate voltage. The electrolyte–graphene interfacial capacitance was measured using capacitance-voltage (C-V) profiling at multiple frequencies using a transistor configuration. The carrier density was then derived based on the analysis of the measured interfacial capacitance; coupling with a conductivity measurement, we further extracted the carrier mobility in graphene.

## 2. Experiments

**Figure 1:**
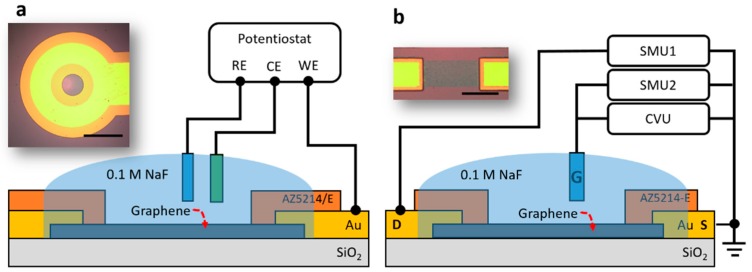
Experimental setup for (**a**) electrochemical impedance spectroscopy (EIS) measurement (scale bar: 1 mm) and (**b**) capacitance-voltage (C-V) profiling and transfer measurement (scale bar: 100 μm).

The schematic experimental setup in an electrode configuration is shown in Figure 1a. The EIS measurement was conducted with a Gamry Interface 1000T potentiostat in a three-electrode structure with a standard Ag/AgCl electrode as the reference electrode (RE) and a gold wire as the counter electrode (CE). The graphene electrode was connected to the working electrode (WE) of the potentiostat. The EIS spectrum was collected over a frequency range from 20 kHz to 0.1 Hz with an AC perturbation voltage of 10 mV rms. The potential of the graphene electrode was swept from −0.5 to +0.5 V vs. Ag/AgCl with a step of 0.05 V.

The schematic experimental setup in a transistor configuration is depicted in Figure 1b. The C-V profiling was conducted with a capacitance-voltage unit (CVU) in a Keithley 4200 Semiconductor Characterization System (SCS). The AC perturbation voltage was set to 10 mV rms. The transfer curve measurement was conducted with two source measure units (SMU) in the Keithley 4200 SCS, and the drain current ($I_{d}$) was measured with a 10 mV load ($V_{\mathrm{ds}}$). In both cases, the gate voltage ($V_{g}$) was modulated using a Ag/AgCl reference electrode and swept from −0.5 to +0.5 V with steps of 1 mV and a scan rate of 10 mV/s.

The microscopic images of the graphene electrode and the graphene transistor are shown in the inset of Figure 1a,b, respectively. Single-layer graphene grown on a Cu foil (ordered from Graphene Supermarket) was transferred onto the silicon wafer with 300 nm oxide and electrically connected using metal contacts fabricated by UV lithography, following the procedure described in our previous reports [3,17]. A photoresist layer was employed to (1) precisely define the graphene area that was exposed to the electrolytes and (2) passivate the metal contacts. A polydimethylsiloxane (PDMS) well with an opening of 5 mm in diameter was placed on top of the chip for the confinement of the electrolyte (0.1 M NaF), and the Ag/AgCl reference electrode was inserted into the PDMS well. The experiments were repeated with three devices.

## 3. Results and Discussion

**Figure 2:**
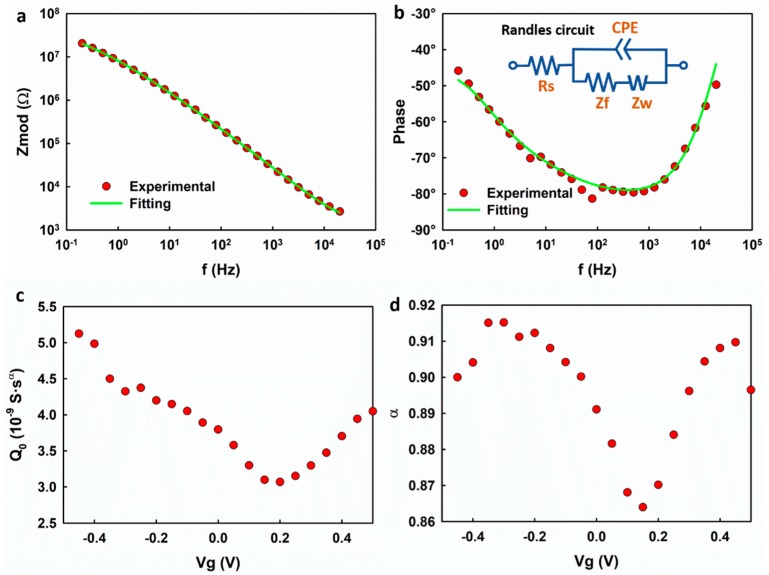
(**a**,**b**) The representative Bode plots of the graphene electrode and the fitting curve based on the Randles circuit; (**c**,**d**) the extracted $Q_{0}$ and α based on the Randles circuit. Note in (b): Rs represents the series resistance, including the access resistance and electrolyte resistance; CPE is the constant phase element; Z_f_ is the faradaic impedance, and Z_w_ is the Warburg impedance.

We first studied the frequency response of the graphene electrode using EIS. Figure 2 shows the representative Bode plot of the graphene electrode. A capacitive regime with a phase shift of ~−80° is observed at 100–1000 Hz. At frequencies lower than 100 Hz, the electrode exhibits a mixed capacitive and resistive response. An equivalent Randles circuit model (inset of Figure 2b) is used to fit the measured impedance, in which Rs represents the series resistance, including the access resistance and electrolyte resistance, CPE is the constant phase element, Z_f_ is the faradaic impedance, and Z_w_ is the Warburg impedance, which is attributed to the diffusion-limited faradaic reaction. The fitting yields a CPE with $Q_{0}$ = 3.8 × 10^−9^ S·s^α^ and α = 0.89 which corresponds to the capacitive regime with a phase shift of ~−80° in the frequency range of 100–1000 Hz. The results suggest that the electrolyte–-graphene interface behaves as a CPE rather than an ideal capacitor.

Using the Randles circuit model, the impedance spectrum of the graphene electrode was analyzed at different gate voltages. To be consistent with the measured results in the transistor mode, in Figure 2c,d, the fitting parameters $Q_{0}$ and α are plotted with respect to $V_{g}$, which equals the negative of the voltages applied to the working electrode. $Q_{0}$ is normalized with respect to the surface area of the graphene. $Q_{0}$ exhibits an ambipolar behavior with a minimum of 3.0 × 10^−9^ S·s^α^ at $V_{g}\approx$ +0.2 V and increases on both sides of the minimum value. Noting that $Q_{0}$ is numerically equal to the capacitance at f = 1 Hz, the result is expected and consistent with previous reports [7,8,10]. The non-zero minimum is attributed to the imperfections in graphene, such as the charged impurities on the SiO_2_ substrate and the defects in the graphene lattice, which could introduce residual carriers [8,10,18]. However, it is interesting that the factor α also exhibits a dependence on the gate voltage, which is unique for typical EDL.

**Figure 3:**
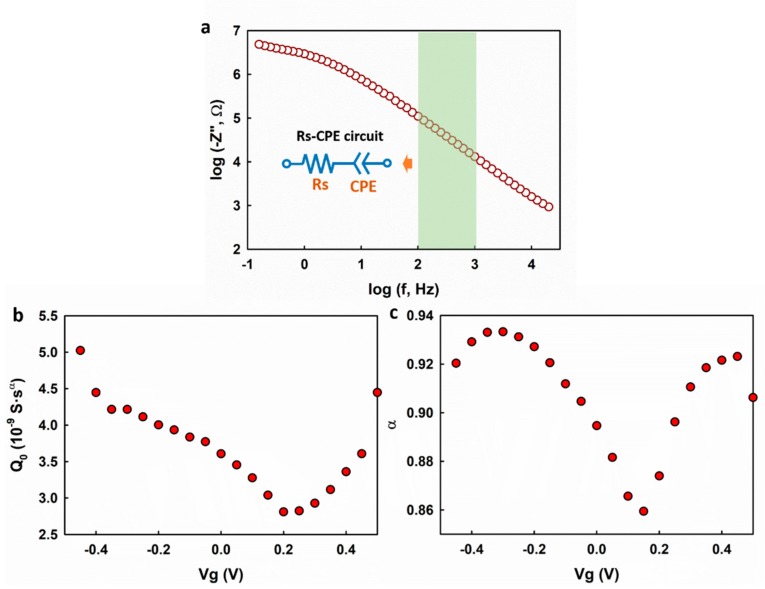
(**a**) $\log\left(-Z^{\prime \prime }\right)$. vs. logf plot with the linear range for fitting highlighted in green; (**b**,**c**) the extracted $Q_{0}$ and α by fitting the impedance spectrum using the Rs-CPE model.

The Randles circuit model was shown to be well fitting to the impedance spectrum. However, it might also introduce uncertainties to the fitting result because a quite few fitting parameters were involved, and this could possibly lead to the observed $V_{g}$-dependence of α. To rule out this possibility, we analyzed the measured impedance spectrum using a simplified electrical circuit model which consists of a resistor and a CPE in a series arrangement as shown in the inset of Figure 3a. For convenience, the simplified model is called the Rs-CPE model. The Rs-CPE model is based on the analysis of the out of phase elements ($Z^{\prime \prime }$) of the impedance spectrum in the capacitive regime. In the absence of charge transfer induced by the Faradaic reactions at the electrolyte–graphene interface, Z_f_ and Z_w_ can be removed leading to a simplified Rs-CPE circuit with the impedance calculated as

(2) $$Z=R_{s}+\frac{1}{Q_{0}{\left(j\omega \right)}^{\alpha }}$$

The out-of-phase ($Z^{\prime \prime }$) component is

(3) $$Z^{\prime \prime }=\frac{-\sin\left(\frac{\pi \alpha }{2}\right)}{Q_{0}\omega^{\alpha }}.$$

Taking the logarithm of both sides, we get

(4) $$\log\left(-Z^{\prime \prime }\right)=\log\left(\frac{\sin\left(\frac{\pi \alpha }{2}\right)}{{\left(2\pi \right)}^{\alpha }Q_{0}}\right)-\alpha \log f$$

As shown in Figure 3a, $\log\left(-Z^{\prime \prime }\right)$. exhibits a good linearity with respect to logf, and this is in agreement with Equation (4). A linear regression analysis was applied in the frequency range of 100–1000 Hz to the impedance spectrum collected at different gate potentials. As shown in Figure 3b,c, both $Q_{0}$ and α exhibit dependence on the gate voltage ($V_{g}$), which is in accordance with the fitting results using the Randles circuit model. The fitting results from the two different models suggest that the observed gate voltage dependence of α is an intrinsic property of the electrolyte–graphene interface.

**Figure 4:**
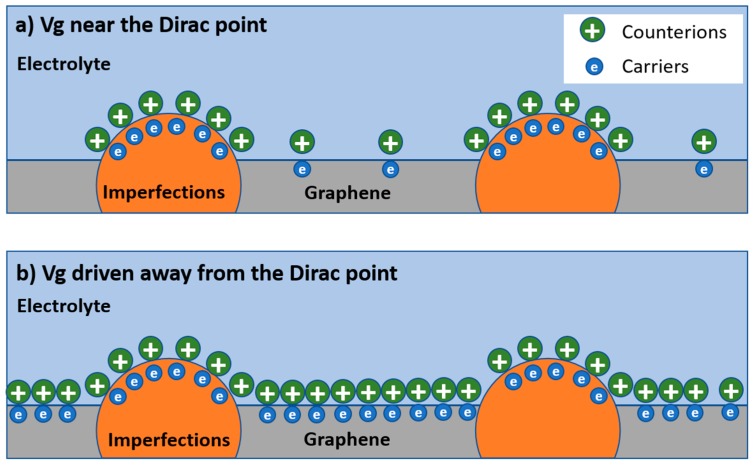
Schematic diagram showing the impact of the imperfection sites on the capacitance behavior of the electrolyte–graphene interface. The distribution of the charges at the electrolyte-graphene interface in the case of (**a**) V_g_ near the Dirac point and (**b**) V_g_ driven away from the Dirac point are demonstrated.

We speculate this unique phenomenon could result from the low DOS in graphene near the Dirac point, which makes α sensitive to the charged impurities on the SiO_2_ substrate and the defects in the graphene lattice. The charged impurities on the SiO_2_ substrate have a significant impact on the transport behavior of the graphene, such as introducing long-range Coulomb scattering, causing local potential fluctuation and electron/hole puddles [8,18], etc. Such effects are well explained by the self-consistent theory [18]. The presence of defects is evidenced by the D peak in the Raman spectrum of the graphene we used in this study (see Supplementary Materials, Note 1). These imperfections exist as local sites with DOS that are different from those in the “good” graphene sites. Considering the single-atom-layer structure and low DOS of graphene at a low energy level, these imperfections could dominate the capacitive behavior of the graphene. A schematic diagram depicting this effect is shown in Figure 4. When the gate potential is low, e.g., near the Dirac point, the carrier density in the graphene is low, and these imperfections dominate the capacitance, resulting in a highly inhomogeneous interface that would give rise to small α values. When the gate potential in graphene is driven away from the Dirac point, the carrier density in graphene is high, and the imperfections are “submerged” by the carriers induced by the gating effect; as a result, the impact of these imperfections is reduced, and a higher α is observed.

**Figure 5:**
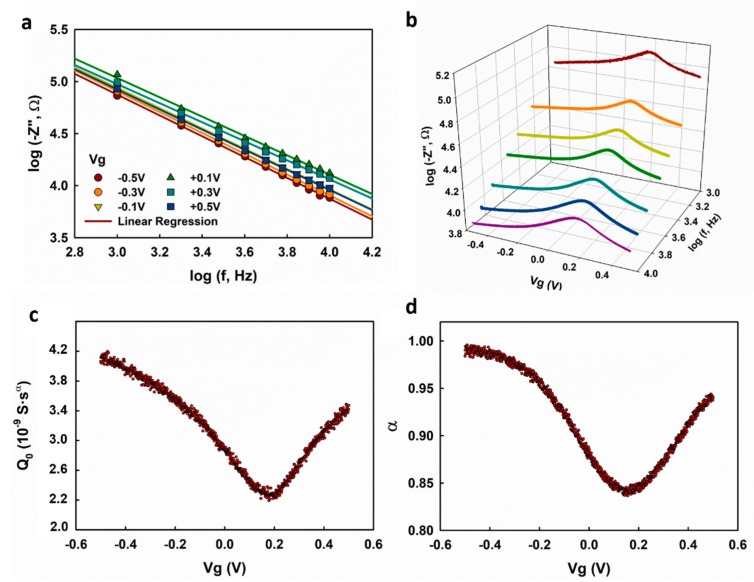
(**a**) $\log\left(-Z^{\prime \prime }\right)$ vs. logf plot measured by frequency sweeping at selected gate voltages; (**b**) $\log\left(-Z^{\prime \prime }\right)$ vs. logf plot measured by gate voltage sweeping at selected frequencies; (**c**,**d**) $Q_{0}$ and α were extracted based on multi-frequency C-V profiling method.

C-V profiling is widely used in the characterization of solid capacitors or transistors [19]. We demonstrate here the characterization of the electrolyte–graphene interfacial capacitance using a multi-frequency C-V profiling technique and show that it can provide more reliable measurement results than EIS. We first measured the frequency response of the graphene transistor at fixed gate voltages, and the obtained $\log\left(-Z^{\prime \prime }\right)$ is plotted with respect to logf in Figure 5a. Good linearity with slopes less than one is observed, suggesting that the graphene transistor works in capacitive regime at 1 k–10 kHz. The impedance of the graphene was then measured at selected frequencies (1, 2, 3, 4, 6, 8, and 10 kHz), and the obtained $\log\left(-Z^{\prime \prime }\right)$ is plotted as a function of logf in Figure 5b. $Q_{0}$ and α are extracted based on the linear regression analysis of $\log\left(-Z^{\prime \prime }\right)$ with respect to logf using Equation (4), as shown in Figure 5c,d. Both $Q_{0}$ and α show dependence on $V_{g}$, which is consistent with the measurement results obtained with EIS, as we discussed previously. Different from the α-$V_{g}$ dependence obtained with EIS, which shows decreases at $V_{g}$ < −0.3 V and $V_{g}$ > +0.4 V (Figure 2d and Figure 3c), the α obtained using multi-frequency C-V profiling exhibits a monotonous increase on both sides of the minimum value. We speculate that the latter result is more accurate because (1) the voltage-sweeping mode gives better continuity for the voltage-dependence measurement, and (2) the C-V profiling measurement takes a much shorter time than EIS, which could effectively circumvent the drift of the electrochemical systems.

Next, we show the extraction of the carrier density in graphene based on the measured interfacial capacitance, which allows us to calculate the carrier mobility in graphene. Based on the linear DOS in graphene, Fang et al. [20] derived the quantum capacitance of graphene as a function of the local electrostatic potential ($V_{ch}$) as follows (see Supplementary Materials, Note 2):

(5) $$C_{Q}=\frac{2e^{3}}{\pi {\left(\hbar v_{F}\right)}^{2}}V_{ch}$$

in which e is the elementary charge; ℏ is the reduced Planck’s constant; $v_{F}$ ~ 10^8^ cm/s is the Fermi velocity of carriers in graphene. Based on the approximation that the total carrier density in graphene is

(6) $$n_{\mathrm{total}}=n_{g}+n_{res}$$

in which $n_{g}$ is the gate voltage induced carrier density; $n_{res}$ is the residue carrier density introduced by the charged impurities. Xia et al. [8] developed a model for the quantum capacitance $C_{Q}$ of graphene (see Supplementary Materials, Note 2 for the derivation of the model):

(7) $$C_{Q}=\frac{2e^{2}}{\hbar v_{F}\sqrt{\pi }}{\left(n_{g}+n_{res}\right)}^{1/2}$$

Considering the series arrangement of the EDL capacitance ($C_{\mathrm{EDL}}$) and the quantum capacitance ($C_{Q}$), the total interfacial capacitance ($C_{\mathrm{total}}$) can be calculated as

(8) $$\frac{1}{C_{\mathrm{total}}}=\frac{1}{C_{\mathrm{EDL}}}+\frac{1}{C_{Q}}$$

**Figure 6:**
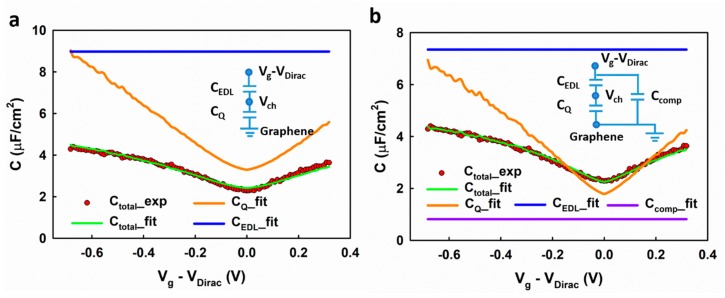
The fitting result of the measured interfacial capacitance using models (**a**) without and (**b**) with a compensating capacitor.

$C_{\mathrm{EDL}}$, $C_{Q}$, and $n_{res}$ can be determined self-consistently by fitting the measured capacitance ($Q_{0}$) using Equations (5), (7) and (8). The experimental result can be fitted using this model, as shown in Figure 6a. However, there is some systematic deviation between the fitted result and the experimental data. For example, at the low voltage range, the fitting results in a slight overestimation of the interfacial capacitance. It is worth noting that a similar deviation is also observed in Xia et al.’s report [8]. We speculate that such deviation could result from the imperfections in graphene, which present a different DOS profile and therefore behave like an extra capacitor in parallel with the graphene sites. As shown in Figure 6b, the experimental results can be better fitted by adding a compensating capacitance ($C_{\mathrm{comp}}$) to the model (inset of Figure 6b). The fitting gives a residual carrier density $n_{res}$ = 1.36 × 10^12^ cm^−2^, which is comparable to previous reports [18,21]. Comparing with the previous report [8], in which the $C_{\mathrm{EDL}}$ was determined by theoretical prediction, our self-consistent fitting method could provide a more accurate result.

**Figure 7:**
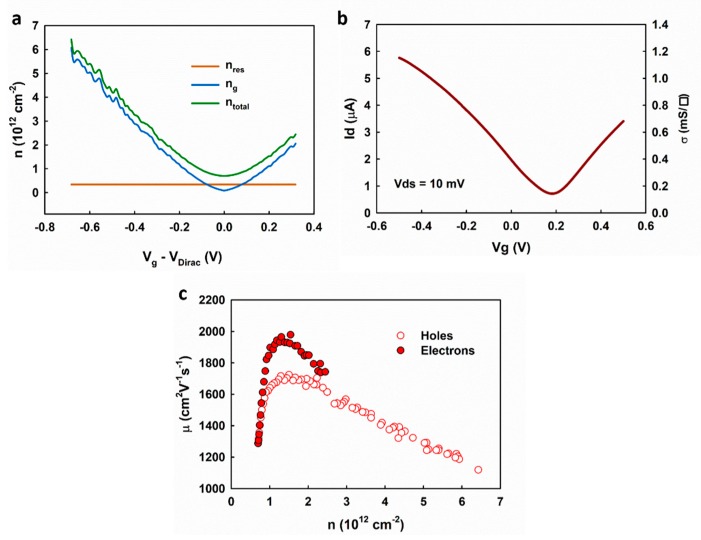
(**a**) The extracted carrier density based on the measured interfacial capacitance, (**b**) the measured transfer curve of the graphene transistor, and (**c**) the extracted carrier mobility.

The carrier mobility can be extracted based on the Drude model:

(9) $$\sigma =en\mu_{e}+ep\mu_{h}$$

in which σ is the sheet conductivity of graphene; n and p are the density of electrons and holes, respectively; $\mu_{e}$ and $\mu_{h}$ are the mobility of electrons and holes, respectively. The carrier densities n and p were extracted based on the fitting results of the measured capacitance (Figure 6b). Based on the mass-action law, we applied a correction to the overall carrier density as

(10) $$np=n_{i}{}^{2}$$

in which $n_{i}$ is the residue carrier density $n_{res}$. As shown in Figure 7a, the total carrier density can be calculated as

(11) $$n_{\mathrm{total}}=\frac{n_{res}{}^{2}}{\left(n_{res}+n_{g}\right)}+n_{res}+n_{g}$$

The sheet conductivity of graphene was calculated based on the transfer measurement of the electrolyte-gated graphene transistor after evaluating the access resistance shown in Figure 7b and Supplementary Materials, Note 3. The extracted carrier mobilities are plotted in Figure 7c, showing a similar dependence on the carrier density as the results given in Reference [22]. The highest mobilities are ~1700 and ~2000 cm^2^V^−1^s^−1^, for holes and electrons, respectively, and decreases when the carrier density is higher than ~1.5 × 10^12^ cm^−2^. The results suggest the transition of the scattering mechanism from the long-range Coulomb scattering (i.e., charged impurities) near the Dirac point to the short-range scattering, which is in agreement with the self-consistent theory [18]. Our results are different from those obtained by the Hall effect measurement [23,24], which shows the highest mobility at the Dirac point and monotonous decrease as the carrier density increases. This is because the Hall effect cannot measure the density of the residual carrier (the Hall voltages induced by the electrons and the holes cancel each other) and thus cause the overestimation of the carrier mobilities near the Dirac point.

## 4. Conclusions

In summary, we study the unique CPE behavior of the electrolyte–graphene interface with both $Q_{0}$ and α showing dependence on the gate voltage. The voltage-dependence of α results from the low DOS of graphene near the Dirac point, which makes α sensitive to the imperfections in graphene from the charged impurities on the substrates and the defects in the graphene lattice. A multi-frequency C-V profiling method is shown to be able to provide a reliable measurement of the factors $Q_{0}$ and α. We further calculated the EDL capacitance and quantum capacitance based on the analysis of the measured interfacial and extracted the carrier mobility in graphene. This study could provide a more accurate understanding of the electrolyte–graphene interfacial capacitance and thus be instructive for the design and analysis of the graphene-based applications that involve electrolytes.

## Supplementary Materials

The following are available online at https://www.mdpi.com/2079-4991/9/7/923/s1, Figure S1: Raman spectrum of the graphene. Figure S2: The plot shows $-Li_{2}\left(-e^{\eta }\right)\;≅\eta^{2}/2$. Figure S3: Transmission line measurement of the sheet conductivity of graphene and the access resistance. (a) The device for TLM study. (b) The transfer curves of each channel with different aspect ratios. (c) The linear regression analysis of the resistance measurement with respect to the aspect ratio. (d) The extracted sheet resistivity of graphene and the access resistance.
