# Supplementary material for: Unique Constant Phase Element Behavior of the Electrolyte–Graphene Interface
Source: Nanomaterials (Basel). 2019 Jun 27;9(7):923. doi: 10.3390/nano9070923 (PMC6669482; doi:10.3390/nano9070923)
Supplement: Supplementary file 1 [file nanomaterials-09-00923-s001.pdf]

Supplemental Information

for

Unique Constant Phase Element Behavior of the Electrolyte-Graphene Interface

Jianbo Sun[1], Yuxin Liu[1,*]

[1] Lane Department of Computer Science and Electrical Engineering, West Virginia University, Morgantown, West Virginia 26506, United States

* Corresponding Author: yuxin.liu@mail.wvu.edu

Note 1. Raman spectrum of the graphene

The presence of the defects is evidenced by the D peak in the Raman spectrum of the graphene which we used in this study.

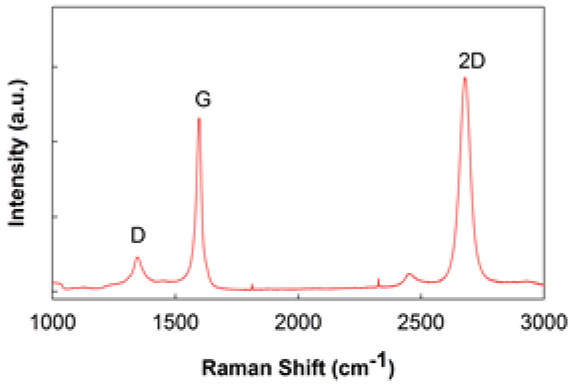

Figure S1. Raman spectrum of the graphene.

Note 2. The derivation of the Electrolyte-Graphene interfacial capacitance model

The dispersion of mobile π electrons in graphene in the first Brillouin Zone (BZ) is given by

$$E(k) = s\hbar v_F |k| \quad \text{(S1-1)}$$

The linear density of states (DOS) in graphene is

$$\rho(E) = \frac{g_s g_v}{2\pi(\hbar v_F)^2}|E| \quad \text{(S1-2)}$$

The carrier density can thereby be calculated as

$$n = \int_0^\infty \rho(E) f(E) dE \quad \text{(S1-3)}$$

where $f(E)$ is the Fermi-Dirac distribution function given by $f(E) = (1 + \exp[(E - E_F)/kT])^{-1}$.

Using the dimensionless variables $u = E/kT$ and $\eta = E_F/kT$, the carrier density can be calculated as:

$$n = \frac{2}{\pi}\left(\frac{kT}{\hbar v_F}\right)^2 \mathcal{J}_1(+\eta) \quad \text{(S1-4)}$$

And

$$p = \frac{2}{\pi}\left(\frac{kT}{\hbar v_F}\right)^2 \mathcal{J}_1(-\eta) \quad \text{(S1-5)}$$

where $\mathcal{J}_j(\eta) = 1/\Gamma(j+1)\int_0^\infty u^j/(1+e^{(u-\eta)})du$ is the Fermi-Dirac integral with $j = 1$ and $\Gamma(\ldots)$ is the gamma function.

The intrinsic carrier concentration is given by

$$n_i = \frac{\pi}{6}\left(\frac{kT}{\hbar v_F}\right)^2 \quad \text{(S1-6)}$$

The carrier density can be rewritten as

$$n = n_i \mathcal{J}_1(+\eta)/\mathcal{J}_1(0) \quad \text{(S1-7)}$$

and

$$p = n_i \mathcal{J}_1(-\eta)/\mathcal{J}_1(0) \quad \text{(S1-8)}$$

respectively.

The total carrier density can be written as

$$Q = e(p - n) \tag{S1-9}$$

The quantum capacitance for graphene $C_Q = \partial Q/\partial V_{ch}$ can be calculated as

$$C_Q = \frac{2e^2 kT}{\pi(\hbar v_F)^2} \ln\left[2\left(1 + \cosh\frac{eV_{ch}}{kT}\right)\right] \tag{S1-10}$$

Under the condition $eV_{ch} \gg kT$, it reduces to

$$C_Q = \frac{2e^2 eV_{ch}}{\pi(\hbar v_F)^2} \tag{S1-11}$$

Recall

$$\mathcal{J}_1(+\eta) = -Li_2(-e^{\eta}) \tag{S1-12}$$

where $Li_2(-e^x)$ is the polylogarithm.

Note that

$$\eta = \frac{eV_{ch}}{kT} \tag{S1-13}$$

For −1 V < V_{ch} < +1 V, −38.7 < η < 38.7. In this range, $-Li_2(-e^{\eta}) \cong \eta^2/2$ (Figure S2).

Therefore,

$$\mathcal{J}_1(+\eta) \cong \frac{\eta^2}{2} = \frac{1}{2}\left(\frac{eV_{ch}}{kT}\right)^2 \tag{S1-14}$$

We obtain

$$n = \frac{2}{\pi}\left(\frac{kT}{\hbar v_F}\right)^2 \mathcal{J}_1(+\eta) \cong \left(\frac{eV_{ch}}{\sqrt{\pi}\hbar v_F}\right)^2 \tag{S1-15}$$

The quantum capacitance can thereby be written accordingly as

$$C_Q = \frac{2e^2}{\pi\hbar v_F} \cdot \sqrt{n} \tag{S1-16}$$

Considering the residue carriers induced by the imperfections in graphene,

$$C_Q = \frac{2e^2}{\pi\hbar v_F} \cdot \sqrt{n_g + n_{res}} \tag{S1-17}$$

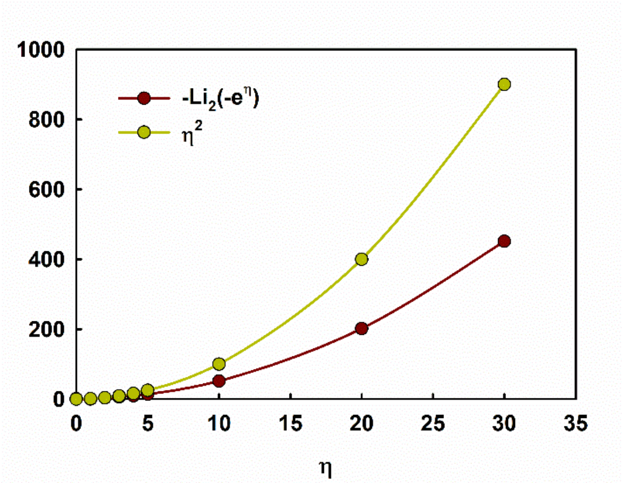

Figure S2. The plot shows $-Li_2(-e^{\eta}) \cong \eta^2/2$

Note 3. Evaluation of the access resistance in the graphene transistor

The sheet conductivity σ of the graphene channel is calculated as

$$\sigma = \frac{I_d}{V_{ds}} \cdot \frac{L}{W} \qquad \text{(S2-1)}$$

where I_d is the drain current; V_{ds} is the voltage applied between the source and drain; W and L are the width and length of the graphene channel, respectively. The measured sheet conductivity σ can be compromised by the access resistance (R_{access}), including the contact resistance ($R_{contact}$) between the metal and the graphene and the resistance of the source and drain electrodes.

To evaluate the impact of the access resistance, we applied a transmission line measurement (TLM). The device used for TLM is shown in Figure S3a. It consists of five graphene channels with different aspect ratios ($\frac{L}{W}$). The total resistance R_{total} is given as

$$R_{total} = \rho_{graphene}\left(\frac{L}{W}\right) + R_{access} \qquad \text{(S2-2)}$$

in which $\rho_{graphene}$ is the sheet resistivity of the graphene. We measured the transfer curves of each graphene channel and the results are shown in Figure S3b. According to the equation S2-2, at a given voltage, R_{total} should be linear with respect to the aspect ratio $\frac{L}{W}$; the slope can be referred as the sheet resistivity of the graphene $\rho_{graphene}$ and the intercept at $\frac{L}{W}$ = 0 is the access resistance R_{access}. As shown in Figure S3c, good linearity is observed. We applied linear regression analysis to the R_{total} with respect to $\frac{L}{W}$ and the $\rho_{graphene}$ and R_{access} are plotted in Figure S3d. The $\rho_{graphene}$ ranges from 0.7 kΩ/□ to 3.5 kΩ/□ as a function of the gate voltage V_g, which is around 5-7 times of the R_{access}. It's worth noting that the access resistance also exhibits dependence on the gate voltage. It can be attributed to the contact resistance between the metal and the graphene; in the vicinity of the Dirac point, the carrier density in the graphene is low which leads to high resistance at the metal-graphene junction.

The aspect ratio of the graphene channel was set to be 2 in our devices because 1) it provides a graphene channel resistance that is around 10 orders higher than the access resistance, and the contribution of the access resistance can be ignored; 2) it provides a practical resistance range for electrical measurement with the current in the range of several μA for a voltage load of 10 mV.

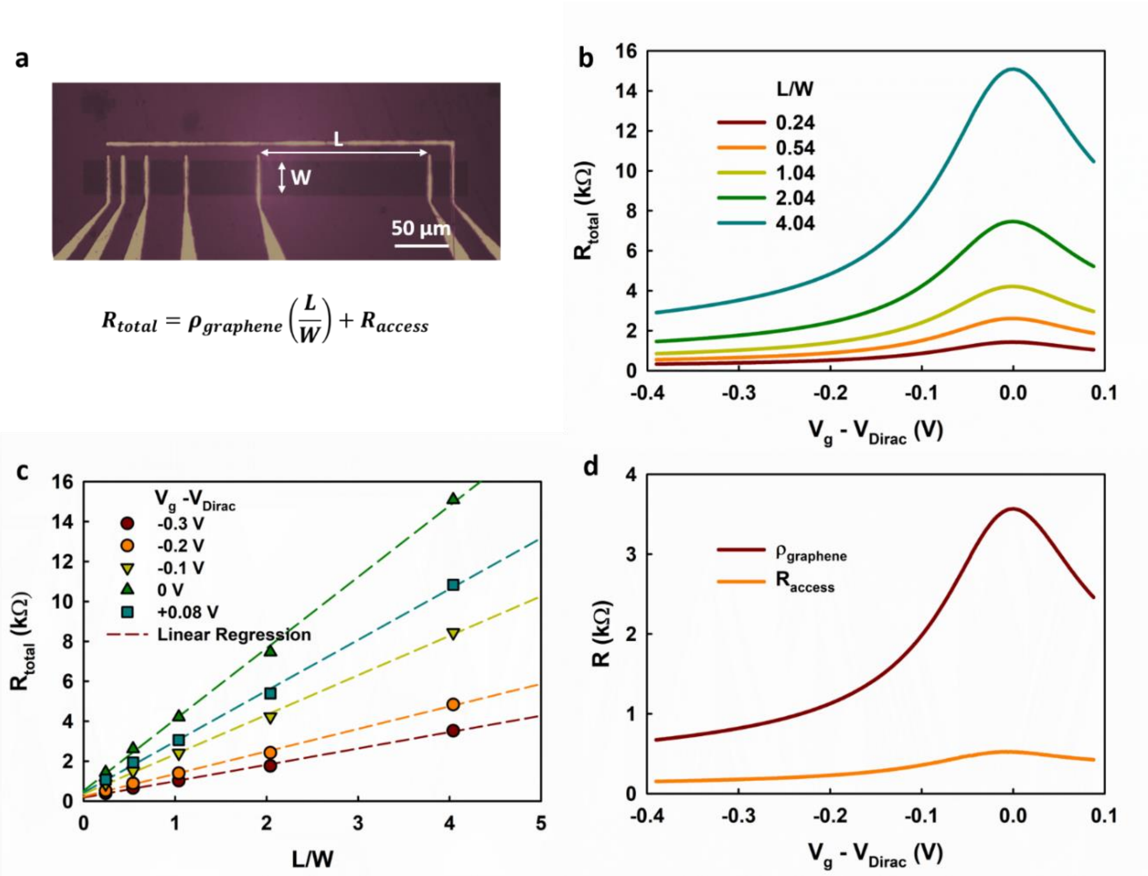

Figure S3. Transmission line measurement of the sheet conductivity of graphene and the access resistance. (a) The device for TLM study. (b) The transfer curves of each channel with different aspect ratios. (c) The linear regression analysis of the resistance measurement with respect to the aspect ratio. (d) The extracted sheet resistivity of graphene and the access resistance.
